# Porcine aminopeptidase N binds to F4^+^ enterotoxigenic *Escherichia coli* fimbriae

**DOI:** 10.1186/s13567-016-0313-5

**Published:** 2016-02-09

**Authors:** Pengpeng Xia, Yiting Wang, Congrui Zhu, Yajie Zou, Ying Yang, Wei Liu, Philip R. Hardwidge, Guoqiang Zhu

**Affiliations:** College of Veterinary Medicine, Yangzhou University, Yangzhou, 225009 China; Jiangsu Co-innovation Center for Prevention and Control of Important Animal Infectious Diseases and Zoonoses, Yangzhou, 225009 China; College of Animal Medicine, Nanjing Agriculture University, Nanjing, 210095 China; College of Veterinary Medicine, Kansas State University, Manhattan, KS 66506 USA

## Abstract

F4^+^ enterotoxigenic *Escherichia coli* (ETEC) strains cause diarrheal disease in neonatal and post-weaned piglets. Several different host receptors for F4 fimbriae have been described, with porcine aminopeptidase N (APN) reported most recently. The FaeG subunit is essential for the binding of the three F4 variants to host cells. Here we show in both yeast two-hybrid and pulldown assays that APN binds directly to FaeG, the major subunit of F4 fimbriae, from three serotypes of F4^+^ ETEC. Modulating APN gene expression in IPEC-J2 cells affected ETEC adherence. Antibodies raised against APN or F4 fimbriae both reduced ETEC adherence. Thus, APN mediates the attachment of F4^+^*E. coli* to intestinal epithelial cells.

## Introduction

F4^+^ enterotoxigenic *Escherichia coli* (ETEC) infections cause neonatal and post-weaning diarrhea (PWD) in piglets. Interactions between F4 fimbriae and specific receptors on the host intestinal mucosa are essential to initiate attachment, colonization, and infection [1, 2]. Some breeds of pigs are resistant to F4^+^ ETEC infection because they lack F4 receptors (F4Rs) [3, 4].

F4 fimbriae are important ETEC virulence factors and exist as three antigenic variants, namely F4ab, F4ac, and F4ad [5]. These three F4 fimbriae are similar, but differ in the *faeG* gene, which encodes the major fimbrial subunit, resulting in different adhesive properties and specificities in attachment to the small intestine [6, 7]. Strains in which *faeG* is deleted exhibit significantly reduced adherence to host cells [8]. Oral administration of F4 fimbriae or FaeG induces a protective mucosal immune response in F4 receptor positive piglets and FaeG mediates ETEC binding to host cells [4, 6, 7]. It seems likely that the major FaeG subunit is not only an essential component of F4 fimbriae but also directly mediates the binding of F4^+^*E*. *coli* [9].

Various potential host receptors for F4 fimbriae have been described, including MUC4, MUC13, MUC20, ITGB5, and TFRC [10–13]. The polymorphic *Xba*I restriction enzyme site in intron 7 of the *muc4* gene has been used as a biomarker to classify an important percentage of piglets as susceptible or resistant to F4^+^ ETEC infections [14–16]. Mucin 4 polymorphisms and their candidate glycoprotein receptors are highly associated with the MUC4-susceptible genotype [17]. However, MUC4 genotypes are not completely associated with F4 ETEC susceptibility and there are likely to be other F4 receptors [18, 19]. Recently, porcine aminopeptidase N (APN) was reported to serve as a receptor protein for F4ac^+^ ETEC [20]. APN, also known as ANPEP and PEPN, is a Zn^2+^ membrane-bound exopeptidase that is highly expressed on the intestinal mucosa [21]. APN can promote intestinal epithelial cell endocytosis in F4Rs piglets and is involved in the induction of mucosal immunity [20]. Here we desired to characterize the interaction between APN and FaeG, to investigate whether modulating APN expression in IPEC-J2 cells could affect ETEC adherence, and to determine whether APN is directly involved in the adherence of F4^+^ ETEC to host cells.

## Materials and methods

### Bacterial strains, antibodies, cell lines, and culture conditions

F4^+^*E. coli* (C83901, O8:K87:F4ab; C83902, O8:K87:F4ac; C83903, O141:K85:F4ad) strains and their respective *faeG* deletion mutants were cultivated in Luria–Bertani (LB) media [8, 22]. Recombinant *E. coli* SE5000 strains carrying the *fae* operon gene clusters, designated as rF4ab, rF4ac, and rF4ad, respectively, were cultivated in LB medium supplemented with ampicillin (100 μg/mL) [23]. Bacteria harboring the pcDNA^TM^6.2-GW/miR-APN-top10 plasmid were cultivated in SOB medium supplemented with 50 µg/mL spectinomycin. All broth cultures were grown with agitation (178 rpm) at 37 °C.

Porcine neonatal jejunal IPEC-J2 cells were grown in RPMI 1640-F12 (1:1) (Gibco) supplemented with 10% fetal bovine serum (FBS, Gibco) at 37 °C in a humidified incubator in an atmosphere of 6% CO_2_. The monoclonal anti-F4 antibody was developed in our lab [24].

### *apn* gene cloning and expression

Total RNA was extracted from jejunum samples of 10-day-old piglets using TRIzol reagent [25]. Reverse transcription polymerase chain reaction (RT-PCR) was performed using Superscript 18080 reverse transcriptase (Invitrogen) with primers (APN-Up1: CGGGGATCCATGGCCAAGGGATTCTAC; APN-Lo1: CCCGCTCGAGTATTAGCTGTGCTCTATG) specific to the porcine APN mRNA (GenBank: NM_214277). PCR products were cloned into pET-28a (+) and transformed into *E. coli* BL21 (DE3) (Novagen) for recombinant expression of APN [26]. The recombinant protein was purified and used to immunize 6-week-old BALB/c female mice to produce polyclonal antiserum specific for APN [27].

### Protein–protein interaction assays

Agglutination assays were conducted as described previously [28]. F4^+^*E. coli* were cultured overnight at 37 °C, diluted with two volumes of PBS after centrifugation, and washed twice with PBS. Bacterial suspensions (10 µL) were applied to glass slides and mixed with APN protein. Visible agglutination within 2 min incubation was considered as positive.

For yeast two-hybrid assays (Y2H, Clontech) [29], pGADT7-FaeG and pGBKT7-APN were constructed and transformed into *Saccharomyces cerevisiae* strain AH109 (Clontech). Positive clones were selected on SD/-Ade/-His/-Leu/-Trp medium and tested for β-galactosidase activity. Yeast transformed with pGBKT7-p53 and pGADT7-T (Clontech) served as a positive control and yeast transformed with pGBKT7-Lam and pGADT7-T (Clontech) served as a negative control.

For pull-down assays, pGEX-6p-1-FaeG F4ab, pGEX-6p-1-FaeG F4ac, and pGEX-6p-1-FaeG F4ad were constructed. These GST-FaeG fusion bait protein expression were induced with 1 mM IPTG at 16 °C for 16 h. GST-APN was loaded on a Pierce™ GST Protein Interaction Pull-Down Kit (Thermo) according to the manufacturer’s instructions [30]. SDS-PAGE and Western blotting were performed to determine whether APN and FaeG interact in vitro. The blots were incubated overnight with either monoclonal antibodies against F4^+^ fimbriae or polyclonal antibodies against APN, and stained using enhanced chemiluminescence (ECL) (Pierce) reagents.

To investigate the role of glycans in the APN-FaeG interaction, in some cases, PDVF membranes were treated with 0–20 mM NaIO_4_ (Sigma-Aldrich) in 50 mM sodium acetate, pH 4.5, at 37 °C in the dark for 30 min–2 h [20, 31, 32]. Membranes were thoroughly with TBST, blocked with a 2% BSA, and then used in Western blotting experiments as described above.

### *apn* knockdown and overexpression cell lines

The *apn* gene was amplified using PCR (APN-Up2 primer: CCCGCTCGAGGAGAAGAACAAGAATGCC; APN-Lo2 primer: GGGCGGATCCTGCTGTGCTCTATGAACCA) (underlined *XhoI* and *BamHI* restriction sites) and then cloned into the pEC129 vector. The woodchuck hepatitis virus post-transcriptional regulatory element (WPRE) was excised from pEC107 and cloned into the *NotI* site of pEC128) [33]. The resultant plasmid was transfected into IPEC-J2 cells using Lipofectamine 2000 Reagent (Invitrogen). G418 (400 μg/mL) was used to select for cells that stably expressed APN [34, 35]. For *apn* gene knockdown experiments, *apn* gene fragments were cloned into the pcDNA™6.2-GW/miR expression vector (Invitrogen) [36]. The resultant pcDNA™6.2-GW/miR-APN plasmid was transfected into IPEC-J2 cells and established cell lines of pcDNA™6.2-GW/miR- *apn* were screened and selected using Blasticidin S Hydrochloride (Blasticidin S HCl, 4 µg/mL).

### Real-time RT-PCR

Total RNA was extracted from IPEC-J2 cells using TRIzol [25]. cDNA was synthesized using PrimeScript™ 1st strand cDNA Synthesis Kit for Perfect Real Time (Takara). Primers (APN-Up3: ATCGACAGGACTGAGCTGGT; APN-Lo3: CAAAGCATGGGAAGGATTTC) were targeted to conserved *apn* sequences. RT-PCR reactions were performed in triplicate, data were normalized to the endogenous reference gene GAPDH (Up1: TGGTGAAGGTCGGAGTGAAC; Lo1: GGAAGATGGTGATGGGATTTC), and analyzed using the 2^−ΔΔCT^ method [37].

### Western blotting

Proteins were harvested in RIPA buffer with PMSF and incubated overnight with polyclonal antibodies against APN. Blots were developed using enhanced chemiluminescence (ECL) (Pierce) reagents.

### Adhesion and inhibition assays

In vitro adhesion assays were performed as previously described [25, 38]. Bacteria (1 × 10^7^ CFUs) were added to a monolayer of about 1 × 10^5^ cells in each well of a 96-well culture plate (Corning, NY, USA) for 1 h at 37 °C (6% CO_2_). Cell monolayer were washed gently three times with PBS and then 0.5% Triton X-100 was added for 20 min. Lysates were serially diluted and spread on LB agar to enumerate adherent bacteria. The experiments were repeated three times.

Both the anti-F4 fimbriae monoclonal antibody and the anti-APN polyclonal antiserum were used for in vitro inhibition assays. Anti-APN polyclonal antiserum at 1:1, 1:10, and 1:100 dilutions was co-incubated with a monolayer of about 1 × 10^5^ IPEC-J2 cells in each well of a 96-well culture plate for 2 h at 37 °C before adding bacteria. The monoclonal antiserum against F4 fimbriae (1:100 dilution) was co-incubated with bacterial suspensions for 30 min at 37 °C (6% CO_2_) with gentle agitation prior to their addition onto the IPEC-J2 cell monolayer. Lysates were serially diluted and spread on LB agar to enumerate adherent bacteria. The experiments were repeated three times.

### Statistical analyses

All analyses were performed using SPSS 16.0 software (SPSS Inc., USA) using t tests. A *p* value of less than 0.05 was considered statistically significant.

## Results

### APN interacts with both F4^+^ fimbriae and with FaeG

**Figure 1:**
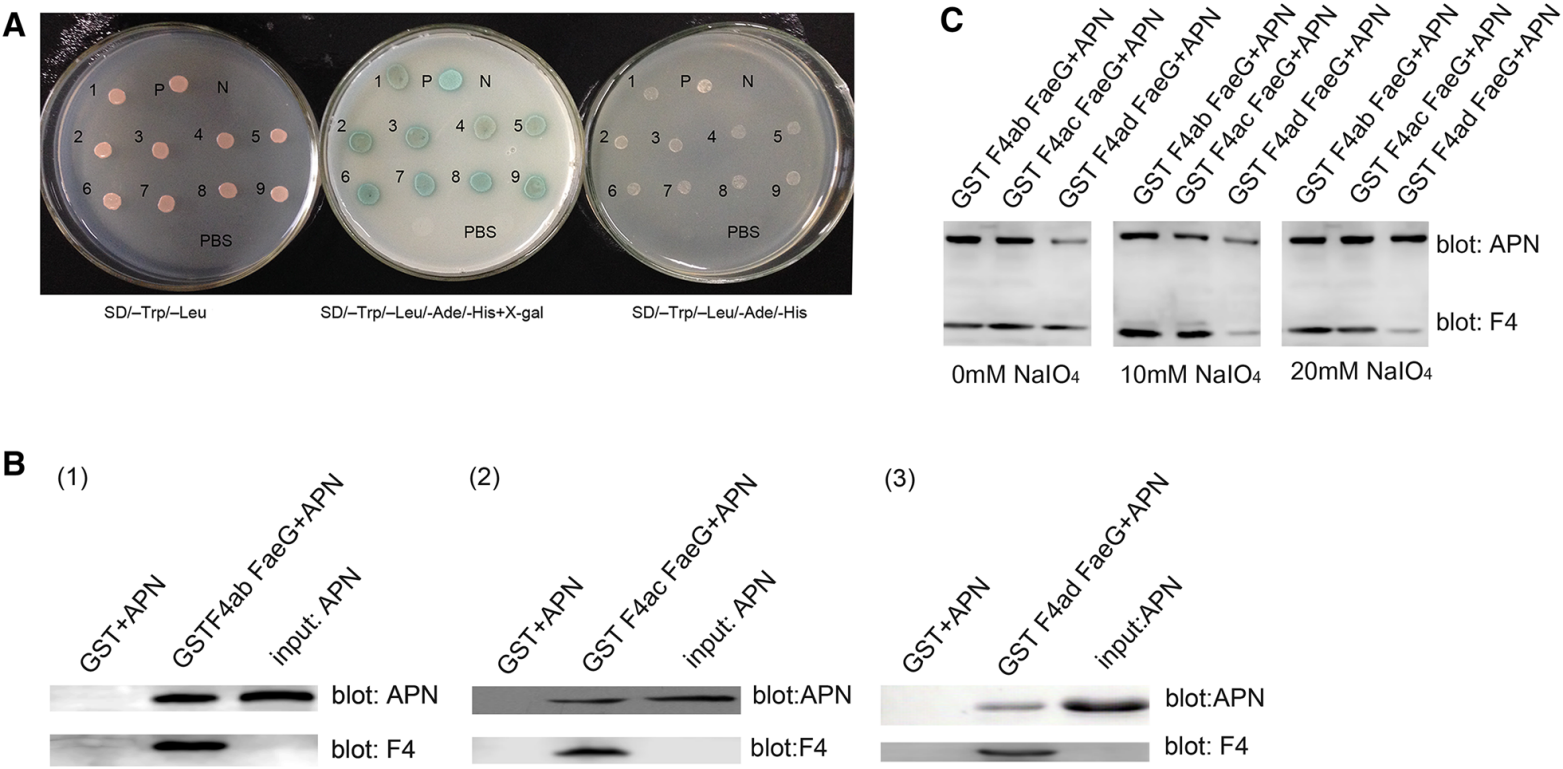
**Yeast two-hybrid (Y2H) and pulldown assays.** **A** Y2H. pGADT7-FaeG and pGBKT7-APN were co-expressed in yeast and positive clones were tested for β-galactosidase activity. Samples 1–3: F4ab FaeG-APN; Samples 4–6: F4ac FaeG-APN; Samples 7–9: F4ad FaeG-APN; N: negative control; P: positive control. **B** GST-pulldown assays. The binding between the recombinant FaeG and APN proteins was studied using the Pierce™ GST Protein Interaction Pull-Down Kit. Western blotting with anti-F4 monoclonal antiserum and anti-APN polyclonal antiserum was used for detection. **C** Metaperiodate treatment. PVDF membranes were treated with either 0, 10, or 20 mM NaIO_4_ before using the membranes in Western blots as described in **B**. Each experiment was repeated three times and representative results are shown.

We first used agglutination assays to test for interactions between APN and F4^+^*E. coli*. Recombinant strains expressing F4 fimbriae had the strongest agglutination, while Δ*faeG* mutants exhibited weak agglutination with APN. Compared with F4ad bacteria, the groups of F4ab and F4ac have a more visible reaction but the difference among three serotypes are not significant (Table 1). Both yeast two-hybrid and pulldown assays were used to determine whether the APN protein binds directly to FaeG. The positive β-galactosidase activities from yeast two-hybrid experiments showed that APN interacted with FaeG when co-expressed in yeast (Figure 1A) and the pulldown results with purified APN and FaeG also demonstrated that APN binds directly to FaeG in vitro (Figure 1B). Treating PDVF membranes to which APN/FaeG pulldown samples had been transferred with metaperiodate (NaIO_4_) did not have significant impact to the results of the APN-FaeG pulldown (Figure 1C).

**Table 1:** **Agglutination assay results between APN and F4** ^**+**^ ***E. coli***

Bacterial Strain	Agglutination with APN^a^
F4ab	+++
rF4ab	++++
F4abΔ*faeG*	+/−
F4ac	+++
rF4ac	++++
F4acΔ*faeG*	+/−
F4ad	++
rF4ad	+++
F4adΔ*faeG*	+/−
SE5000	−
DH5α	−

^a^++++ = nearly 100% agglutination; +++ = turbid background with intermediate agglutination; ++ = obvious but not strong agglutination; +/− = insignificant agglutination; − = no agglutination.

### F4^+^ binding to IPEC-J2 cells differing in APN expression

**Figure 2:**
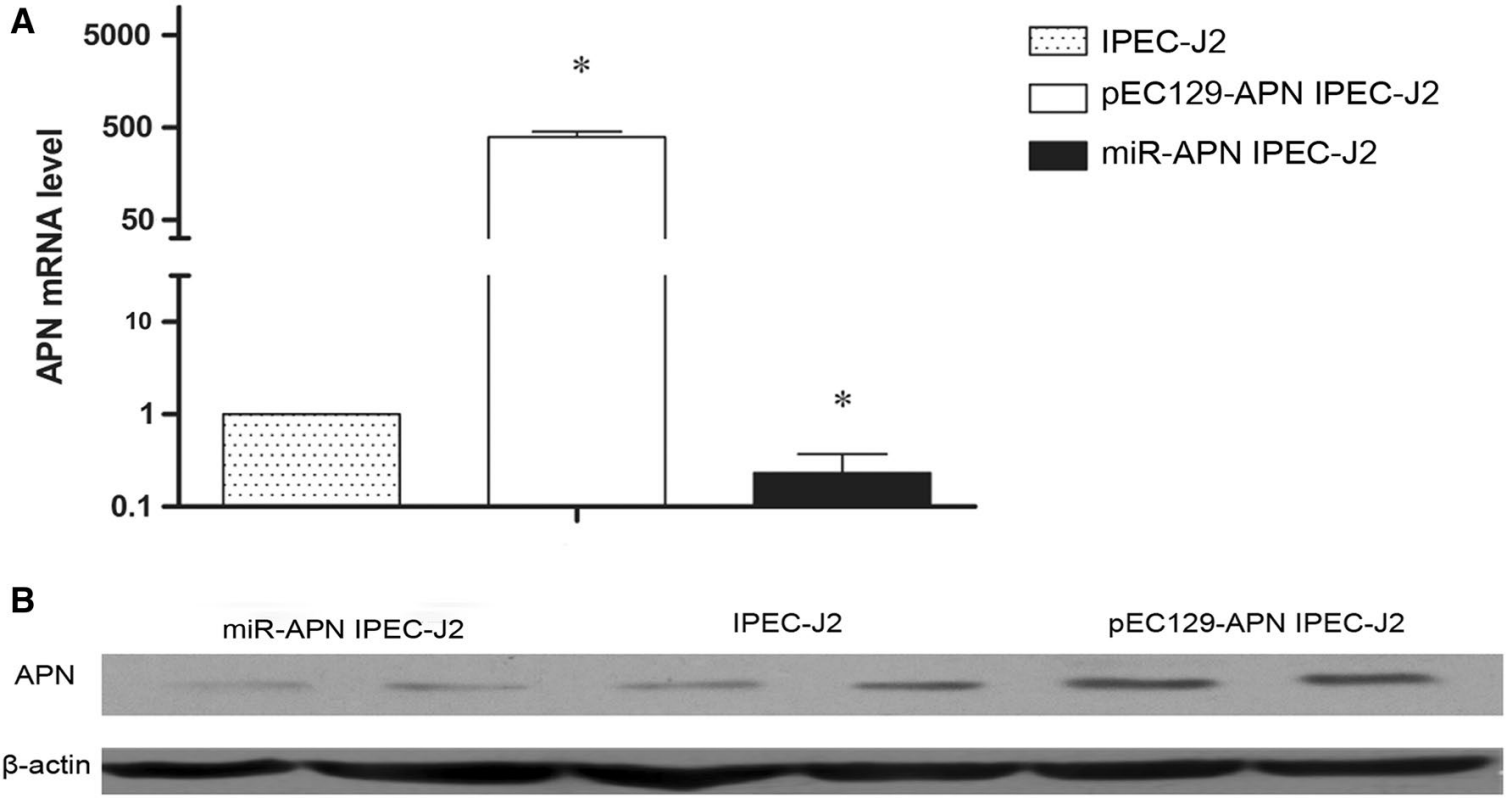
***apn*** **knockdown and overexpression in IPEC-J2 cells.** **A** RT-PCR. *apn* mRNA levels in pEC129-APN-IPEC-J2 cells, pcDNA™6.2-GW/miR-APN-IPEC-J2 cells, and IPEC-J2 cells were quantified using RT-PCR and normalized to *gapdh* expression. The asterisk indicates a statistically significant difference in expression when compared to the original cells (*p* < 0.05). **B** Western blotting. Proteins from IPEC-J2, pEC129-APN-IPEC-J2, and pcDNA™6.2-GW/miR-APN-IPEC-J2 cells were harvested in RIPA buffer. Blots were incubated overnight with polyclonal antibodies against APN and stained with enhanced chemiluminescence (ECL) (Pierce) reagents. Each experiment was repeated three times and representative results are shown.

**Figure 3:**
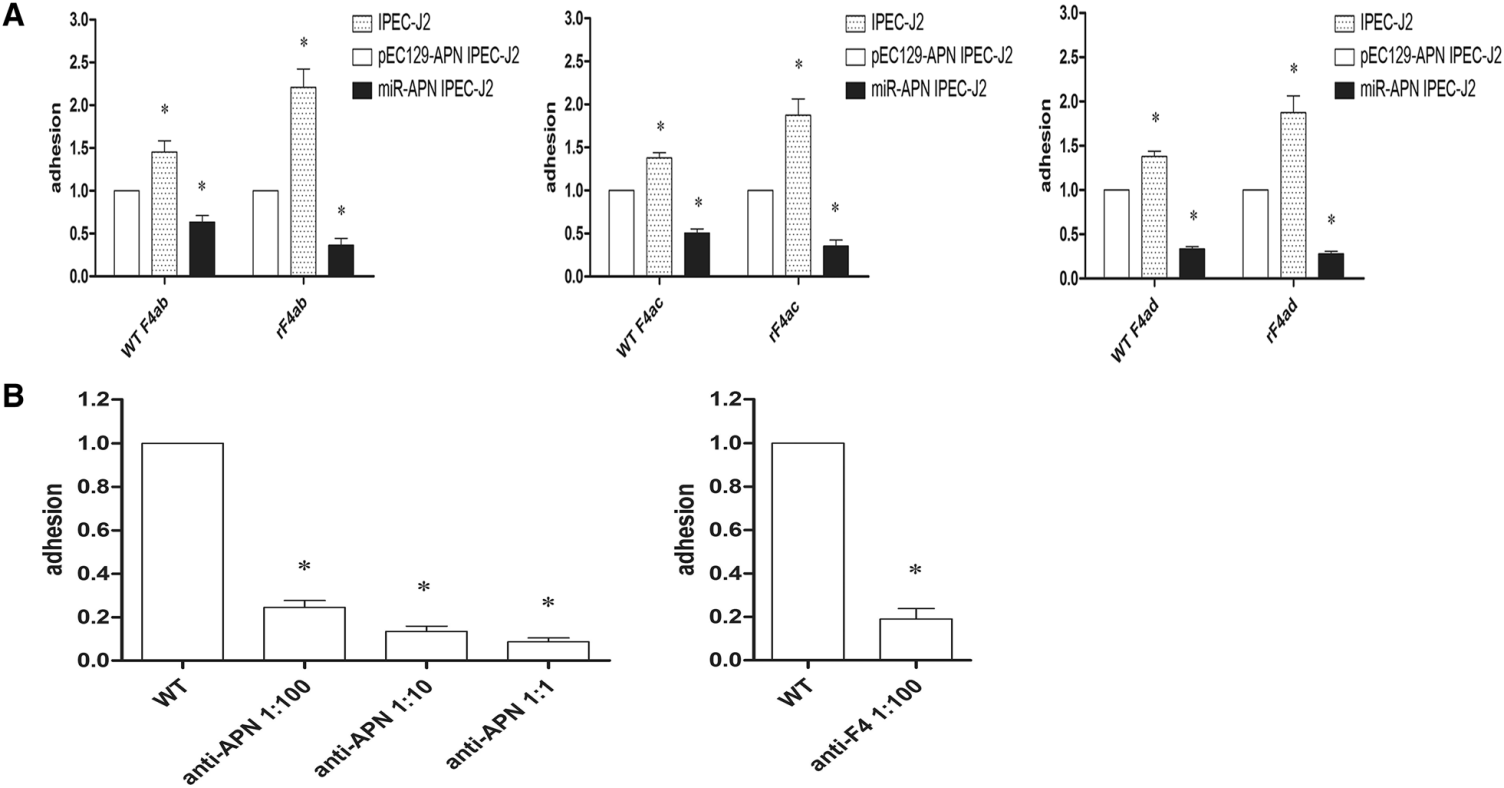
**Modulating APN expression affects ETEC adherence.** **A** Adhesion of F4 *E. coli* strains to IPEC-J2 cells. Bacterial adherence to the original IPEC-J2 cell line was normalized to 100%. **B** In vitro inhibition assay. Adherence of F4^+^ ETEC to IPEC-J2 cells after pre-incubation with anti-F4 fimbriae monoclonal antiserum (1:100 dilution) or with anti-APN polyclonal antiserum (1:1, 1:10, 1:100 dilutions). The adhesion of the untreated samples was normalized 100%. The experiments were repeated three times and data are expressed as mean ± standard deviations (*p* < 0.05).

To evaluate the potential involvement of APN as an F4^+^*E. coli* receptor, we knocked down APN expression in IPEC-J2 cells using pcDNA™6.2-GW/miR-APN. We observed a substantial reduction in APN expression as determined by using RT-PCR (Figure 2A) and Western blotting (Figure 2B). The adhesion of F4 ETEC to IPEC-J2 cells transfected with pcDNA™6.2-GW/miR-APN was significantly reduced (Figure 3A).

The cell line, pEC129-APN-IPEC-J2, that over-expresses APN was also constructed and characterized by using RT-PCR (Figure 2A) and Western blotting (Figure 2B). The adhesion of F4 ETEC to the pEC129-APN-IPEC-J2 cells was substantially increased, as compared with adhesion to the original IPEC-J2 cells (Figure 3A). The addition of both APN polyclonal antiserum and an anti-F4 fimbriae monoclonal antibody to IPEC-J2 cells also reduced ETEC adhesion (Figure 3B).

## Discussion

F4^+^ ETEC infections cause diarrhea in newborn and weaned piglets and fimbriae-mediated adherence to porcine intestinal cells is an initial step in the infection process [39]. The major fimbrial subunit FaeG directly mediates the binding of the three F4 variants to different host receptors [8, 23, 41]. The functional site of the F4ab FaeG subunit is contained within amino acids (AAs) 140-145 and 151-156, while AAs 147-160 dictate binding capacity for F4ac FaeG [40, 41]. The F4ad FaeG subunit interacts with a minimal galactose binding epitope via its D’-D’’-α1-α2 binding domain within AAs 150-152 and 166-170. This D’-α1 loop differs among the FaeG variants and results in their different structural and adhesive properties [42].

While it is known that F4 fimbriae receptors on the gut epithelium determine susceptibility to F4^+^ ETEC, the identity of these receptors is still under active investigation [3, 43, 44]. Polymorphisms in intron 7 of the *MUC4* gene have been used to classify an important percentage of piglets as susceptible or resistant to F4 ETEC [16, 17]. Although Ren et al. and Zhou et al. both found that susceptibility/resistance toward ETEC F4ac is conferred by the *MUC13* gene in pigs, Schroyen et al. reported that *MUC13* and *MUC20* gene expression are not related to ETEC F4ac susceptibility in piglets, and Goetstouwer et al. recently confirmed that MUC4 and MUC13 are not completely associated with F4ab/ac ETEC susceptibility [10, 11, 13, 18].

Several glycoproteins and glycolipids isolated from porcine intestinal cells have been studied for their potential to act as F4 receptors, such as GP74 (TF), IGLad (intestinal neutral glycosphingolipid), and IMTGP (intestinal mucin-type glycoprotein) [43, 45, 46]. However, the functions of these potential receptors are not well characterized.

Here we characterized a newly described receptor for F4^+^ fimbriae, APN, which also serves as a receptor for the transmissible gastroenteritis virus (TGEV), porcine epidemic diarrhea virus (PEDV), and coronavirus [20, 47, 48]. APN is particularly highly expressed in the intestinal mucosa and is also associated with the MUC4 susceptible genotype [17, 21]. APN was recently described as a potential receptor for F4ac^+^ fimbriae; variations in the α2-3,6,8 sialic acid binding site of APN result in reduced binding of F4 fimbriae and binding of F4 fimbriae to APN results in clathrin-mediated endocytosis of the fimbriae [20]. Goetstouwers et al. reported that there are no genetic polymorphisms or expression differences in the ANPEP gene that have been associated with F4 ETEC susceptibility and hypothesized that differences in F4 binding to ANPEP are due to modifications in carbohydrate moieties [20, 49].

We found that IPEC-J2 cells express APN and that F4 *E. coli* was able to adhere to IPEC-J2 cells in an APN-dependent manner. Pre-incubation with APN polyclonal antiserum and anti-F4 fimbriae monoclonal antibody both reduced ETEC adherence to IPEC-J2 cells. Results from Y2H and pulldown assays also showed that FaeG binds directly to APN. We did not find an impact on APN-FaeG binding after treating samples with metaperiodate (NaIO_4_), suggesting that, at least under our in vitro conditions, APN glycosylation does not play a significant role in FaeG binding. However, the molecular details regarding APN-FaeG interactions and their roles in ETEC adherence await further experimentation.
